# Supplementary figures and images for: Teaching a new mouse old tricks: Humanized mice as an infection model for Variola virus
Source: PLoS Pathog. 2021 Sep 21;17(9):e1009633. doi: 10.1371/journal.ppat.1009633 (PMC8454956; doi:10.1371/journal.ppat.1009633)

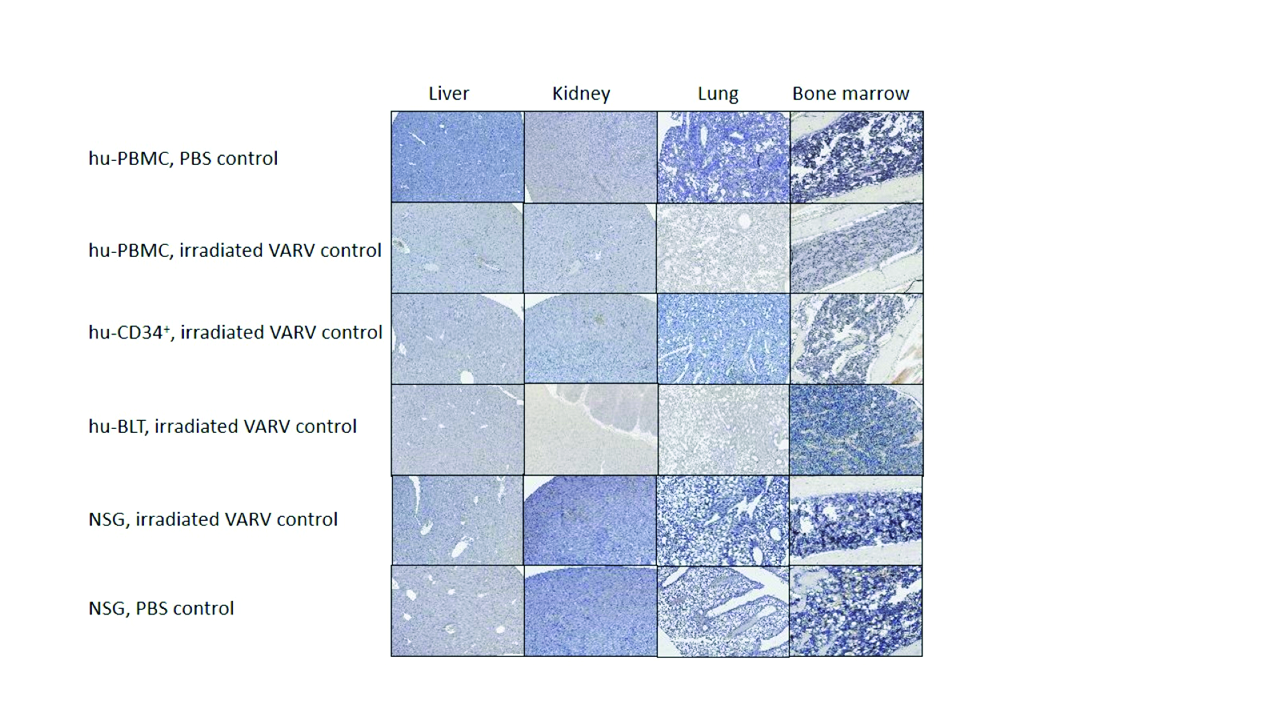

Supplement: S1 Fig — No VARV immunostaining (viral antigen labeling in red) is present in liver, kidney, lung, or bone marrow from hu-PBMC mouse inoculated with PBS only (top row, hu-PBMC-1, day of death 21 dpi); hu-PBMC mouse inoculated with gamma-irradiated VARV (second row, hu-PBMC-3 day of death 13 dpi); hu-CD34+ mouse inoculated with gamma-irradiated VARV (third row, hu-CD34+-1); hu-BLT mouse inoculated with gamma-irradiated VARV (fourth row, hu-BLT-1, day of death 21 dpi); NSG mouse inoculated with gamma-irradiated VARV (fifth row, NSG-11, day of death 21 dpi); NSG mouse inoculated with PBS only (bottom row, NSG-16, day of death 21 dpi). Original magnifications: liver, kidney, lung (x40); bone marrow (x100). (TIF) [file ppat.1009633.s001.tif]

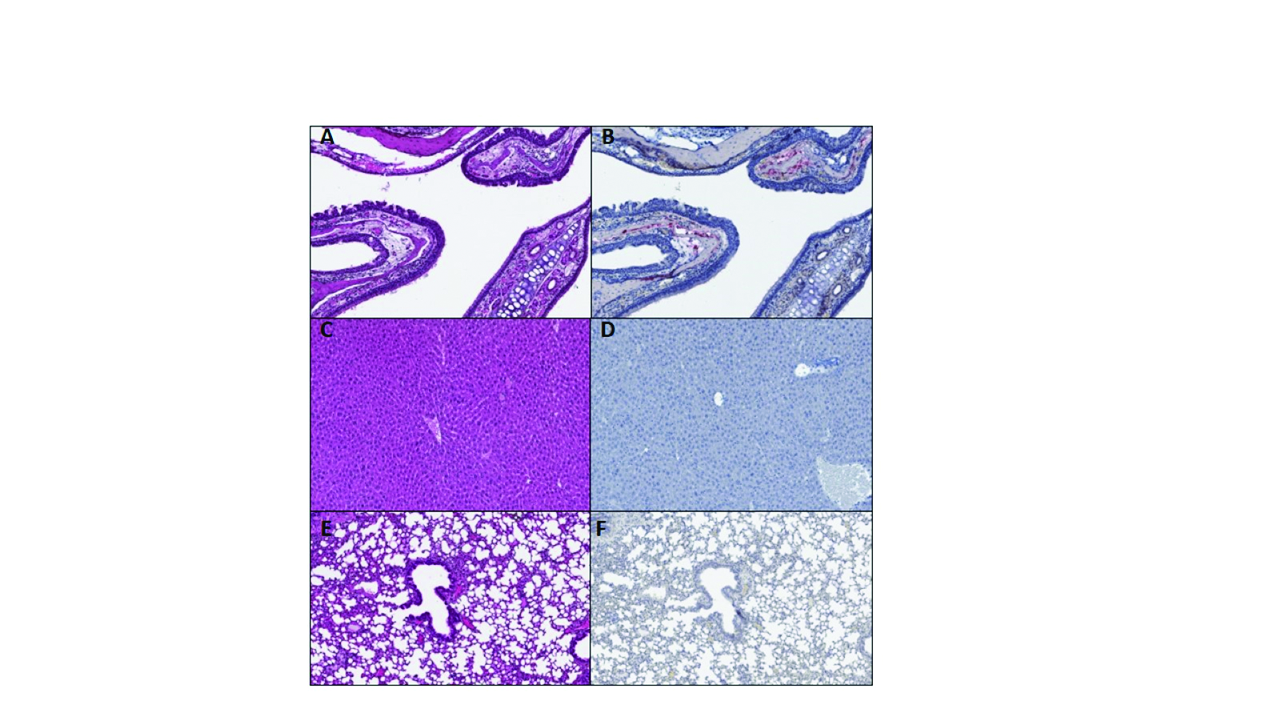

Supplement: S2 Fig — A, B: Nasal tissue with mild inflammatory changes and immunostaining of VARV (NSG-9, high dose, day of death 21 dpi). C, D: Liver without histopathologic changes or immunostaining of VARV (NSG-9, high dose). E, F: Lung without histopathologic changes or immunostaining of VARV (NSG-5, low dose). A, C, E (hematoxylin-eosin); B, D, F (VARV immunohistochemistry, viral antigen labeling in red). Original magnification: all images (x100). (TIF) [file ppat.1009633.s002.tif]

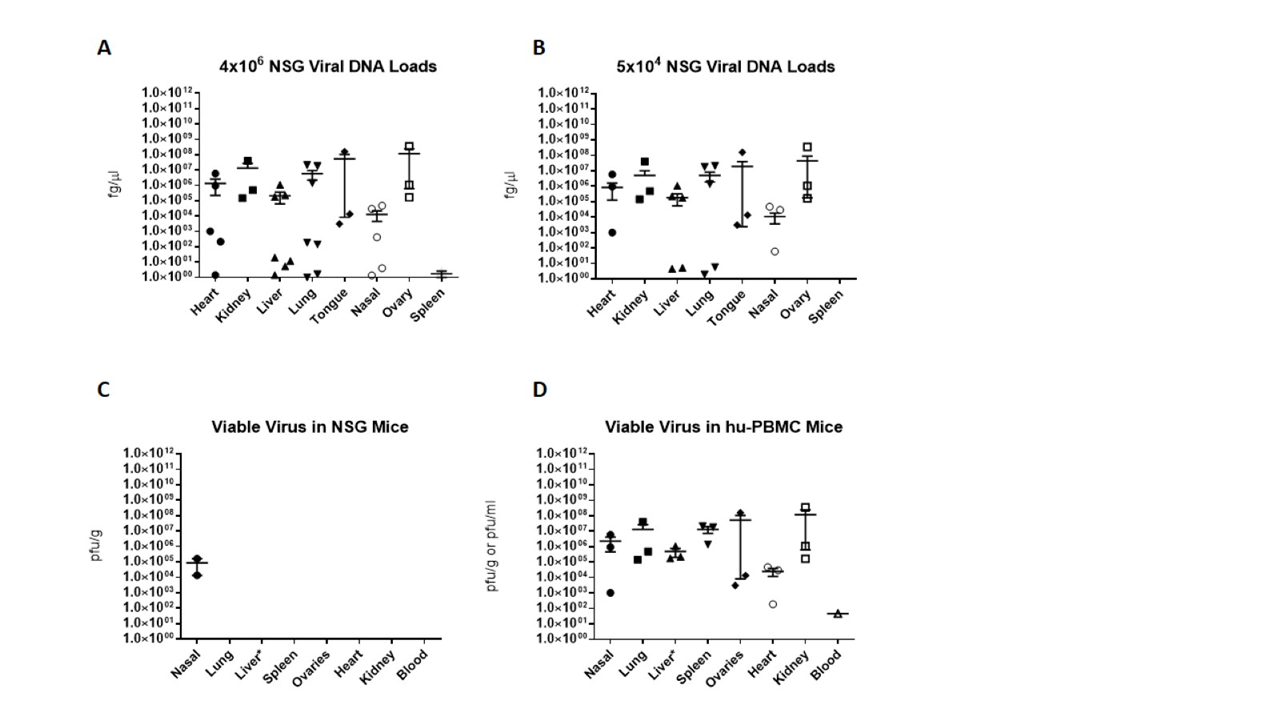

Supplement: S3 Fig — NSG mice were inoculated with 4x10^6^ or 5x10^4^ pfu for a high and low dose group (n = 5 per group). Three hu-PBMC mice were also included and challenged with 4x10^6^ pfu to serve as positive controls. On 21 dpi, animals were euthanized. (A,B): Several necropsied tissues from NSG mice contained low levels of viral DNA mainly the lung, liver and nasal cavity in both challenge groups. (C) Only two nasal cavities, one animal from the low (NSG-1) and one from the high dose group (NSG-6) contain viable virus. An * indicates one or more of that sample had cell culture monolayer destroyed or plaques were present but below the LOD for this assay. (D) In contrast, virus spread systemically through the n = 3 hu-PBMC mice leading to clinical signs and one animal had to be euthanized on day 19 due to a clinical score of 10; high loads of viable virus were found throughout most tissues tested from the hu-PBMC mice. (TIF) [file ppat.1009633.s003.tif]
