# Supplementary material for: Teaching a new mouse old tricks: Humanized mice as an infection model for Variola virus
Source: PLoS Pathog. 2021 Sep 21;17(9):e1009633. doi: 10.1371/journal.ppat.1009633 (PMC8454956; doi:10.1371/journal.ppat.1009633)
Supplement: Attachment — Submitted filename: Answers to Reviewers.docx [file ppat.1009633.s004.docx]

01/26/21

To the Editor and reviewers,

Thank you for the review of our manuscript (“Can you teach a new mouse old tricks? Humanized mice as a model for *Variola virus*”) and for providing comments that are very helpful in improving the quality. We have added responses to each comment below in a table format, and addressed using track changes within the manuscript.

Kind regards,

Christina L. Hutson PhD, MS

Lead, Virus-Host Molecular Interactions Team

Microbiologist

Poxvirus and Rabies Branch

Division of High Consequence Pathogens and Pathology

Centers for Disease Control and Prevention

1600 Clifton Road

Atlanta GA 30333

Building 23 Fourth Floor Office 135

MS- H23-4

404-639-1081

| **Comments** | **Response to Reviewer Comment** |
| --- | --- |
|  |  |
| Even the virus loads are presented on a per-animal basis instead of being compiled in a way that makes basic statistical parameters easy to assess mean/median and a representation of error or distribution. | Changes made |
|  |  |
| unless the virus infectivity titrations in figure 5 have only been conducted once without replicate assays. | Done- Added to Methods |
|  |  |
| Further discussion on the component of the human immune system recreated in hu-PBMC mice (showing mild disease) as compared to hu-BLT and hu-CD34+ mice (showing severe disease) would be interesting and maybe informative.More discussion on the infection of mouse tissues by VARV in this model and the existing evidence of the susceptibility of mouse cells to VARV would be helpful. This would emphasize the role of human immune cells in the establishment of a systemic infection in these mice. | Added additional information within the discussion. |
|  |  |
|  |  |
| Negative control staining of anti-VARV antibodies against uninfected mouse tissues should be shown. | Supplemental Figure S1. Both negatives (uninfected mouse tissues w/ VARV antibodiy and + VARV mice with hyperimmune serum that's not specific). Added Figure S1 with uninfected mice and adjusted numbering of figures throughout. |
|  |  |
| 1. Title. It is established that mice can be engineered to become susceptible to infection by different viruses to which normally they are resistant. This was shown first with polio virus and more recently there are other examples as mentioned in the introduction. Given this, the question posed in the first half of the title is already answered. So the title should be shortened by removal of the first part. | We appreciate the reviewer's opinion but feel the title is appropriate as these mouse strains have not previously been evaluated for VARV |
|  |  |
| 2. In several places the paper is drafted for a USA audience who may understand what a “select agent” is and what CDC and FDA are. For the benefit of the international readership of PLOS Pathogens could these terms be defined / explained more fully? e.g. US FDA, US CDC (there is also a CDC in Europe) and the nation from which terms such as “select agent” and “select agent regulations” derive be defined. | We have changed throughout as requested. |
|  |  |
| 3. The abstract concludes that the model described may be useful for 3 things: testing MCMs for smallpox, identifying why VARV is a pathogen only of humans, and provides samples for understanding the human immune response to VARV infection. This reviewer agrees with the first point, but not the latter two and recommends these should be deleted. | We understand the reviewer’s recommendation, and have deleted two points as requested from abstract. Added a bit of those to end of conclusions. |
|  |  |
| 4. Lines 54-55. It is not clear what “current recommendations” are referred to. Suggest deletion of “improves upon current recommendations by”. | Changed wording to clarify |
|  |  |
| 5. Line 65-66. Reconstruction of horsepox virus. It may be misleading to suggest that horsepox virus (which is considered a strain of VACV) is closely related to VARV. Although all OPXVs are closely related, within the OPXV genus VACV and VARV are quite distinct and taterapox virus and camelpox virus are closest to VARV. | Changed wording as requested. |
|  |  |
| 6. Line 96. define IACUC | Defined |
|  |  |
| 7. Line 114. Specify if anesthesia was used. Similarly, line 129, define “5% inhalation anesthesia” – inhalation of what at 5%? | Done-changed wording to clarify |
| 8. Line 155. “As” is missing from this sentence. | Corrected. |
| 9. In general the figure legends i) do not provide enough detail of what was done or what is shown, and ii) contain too much interpretation of material presented, which should instead (mostly) be contained in the results narrative. | We have reworded most figure legends as requested. For the histopath legends, we feel descriptive wording is appropriate for the histopath to explain what is there since presumably most people reading this won't be pathologists. |
| 10. Fig. 1. The labeling and presentation of this figure needs improvement. Data presented in panels A and B are also included in C and D. So delete A and B. Please relabel figure with increased font size. In the legend define what high and low mean - this could easily be included on the figure itself. Change “survivorship” to “survival” (in text also) Day = day post infection. | We have changed the legend to clarify the figure as requested. |
| 11. Lines 178 – 184. If this animal was not infected correctly it should be removed from all data presented and the group size redefined. | Clarified that the animal was excluded from analysis. |
| 12. Line 194. Please explain what “gamma-irradiated control hu-PBMC mouse” is. There is no mention of gamma-irradiation in the Fig. legend. | Clarified in paper and in the figure legend as requested. |
| 13. Fig. 2. Please re-write legend to describe clearly which animals these samples are from, what dose of virus was given, the day post infection the samples were harvested, and what VARV positive staining looks like. In the histological images please use arrows to point out the features to which the text refers. Ditto Fig. 3. In Fig. 3 arrows are referred to, but were not visible to this reviewer. Specify which panel(s) they are in. | We have inserted the animal IDs and if they were in low or high dose group and the dpi for animals. We indicated in each legend that "viral antigen labeling in red". There were already arrows in Figure 3 in the ppt - We added letters to each image and specified which ones have arrows. We added a few more annotations - open arrows and arrowheads to clarify. We cannot annotate more changes in fig 3 because the observations are more diffuse. |
| 14. Line 254 Define “select tissues”. | Defined. |
| 15. Fig. 5. Define “processed for viral culture”. Plaque assay, or something else? The number of replicate samples, the number of times the plaque assay (or other assay) was repeated need defining, and error bars should be added. A statistical analysis should be included to justify what is “significant”. | The processing of tissue samples is detailed within (Sample processing and DNA extraction) method section. We have described in the methods additionally the number of replicates as requested , and added error bars to the figure. What is considered significant is in the statistical methods section. |
| 16. The legend to Fig. 5 defines a *. But no * is visible in the figure. Define “LOD”. | It is visible on our version (next to some of the sample names (i.e. *lung). |
| 17. Line 282. Explain what “Gross findings were absent during necropsy” means. | clarified |
| 18. Line 297. Consider changing “extremely” to “increasingly”. | changed |
| 19. Lines 318-321. Sentence repetition. This reviewer feels this sentence overstates what has been shown. The report shows that these mice can be infected with VARV and develop a disease with some similarity to human smallpox. The report has not shown that this model will be useful for studying VARV infection, or potentially features of the human immune response to infection. On the other hand, it is reasonable to propose that this model may be useful for evaluating the effectiveness of drugs against smallpox. Similar comment for last part of discussion. | Thank you, we have corrected and tweaked the sentence as well as the discussion per the recommendation. |
| 20. In the discussion, the claim that the model has features similar to hemorrhagic smallpox, or ordinary smallpox, is not well founded base on data presented, are unnecessary and should be deleted. This will help reduce the discussion, which is rather too long. | We feel the pathologic findings from this model does resemble that described for humans and during the NHP studies. Namely "Pertinent features include hepatosplenic, lymphoid, and hematopoietic necrosis, with widespread VARV antigen and nucleic acid detection in these and other tissues, and the uniform presence of bacteremia with a variety of gram-positive cocci at the time of death. Systemic bacterial infections due to gram-positive cocci are a common feature of fatal human smallpox[8, 22, 23], and were also uniformly seen in intravenously inoculated macaques that developed hemorrhagic disease" |
| 21. Line 372 typos “pathogenby compareing” | Corrected. |
| 22. Lines 371-83. Discussion on why VARV is a pathogen only of humans should include consideration of gene loss during VARV evolution, see Science, 2020 DOI: 10.1126/science.aaw8977. The focus on the O1L gene is more about why other related viruses do not infect man, and so might also be deleted to shorten the discussion. | Added additional information to this paragraph as suggested. |
|  |  |
| Reviewer #2: 1) The paper is not written in a conventional way in which the results section has a narrative so that readers can easily follow what has been done. Rather this information has to be obtained by reading the Materials and Methods before the Results. This is unwieldy and will be more so if the manuscript was formatted with the Methods after the Discussion as per the journal style. Further, the data on the numbers of mice with different findings is put in supplementary tables, when they are key to understanding what is happening across each group of mice. These need to be in the main paper. In the second table the groups should be separated by dose as they are in the first table. | We have added additional background information on the huMouse strains in the intro and made these additional changes to clarify: We have changed wording within figure legend to be more clear. 2) In M&M clarified that n= means the group size. **3**) Moved Supp 2 Table into main manuscript. 4) Table 2 broken apart by dose group except for NSG b/c no findings. |
| 2) The issue of just how many mice were in each group is made more difficult to follow because the authors use “n” to indicate the number of mice with a particular finding and not the total group size as defined conventionally in statistics. Further statements about the frequency of signs of illness that aggregates this across the three different types of humanized mice is not helpful in understanding whether one model would be preferred over another. As noted above, key data like the virus titers need to be presented in a more conventional group-wise manner. Individual data can and should still be shown (as points), but with the titers from the same organ (and experimental group) plotted together. This would allow comparisons across organs or mouse types. | Moved down supplemental tables to main part of paper to help reader better understand the findings for each mouse strain. We have updated titers from organs as requested within Figure 5. |
| 3) A key feature piece of information that is required to truly understand this model is the extent to which virus replication is supported in mouse cells versus human cells. This is accessible to some extent from the histopathology, immunohistochemistry and descriptions, that find in at least some cases that VARV is indeed replicating in mouse tissues (e.g. to cause hock lesions), but is not addressed systematically. **For example it would be interesting to know how much of the virus titer in organs may have been contributed by the human cells in transit. Some assessment of this could be made by qPCR for human markers to see is virus levels correlated with human cell levels. Perhaps mice were perfused to limit this complication, but this is not mentioned. In any case, adding more data that explores this issue would add more broad scientific interest to the paper.** | We have added additional information in the discussion. We feel that doing a serial sacrifice study, where early time points are taken, would be help in addressing the human cell(s) needed for infection. |
| 4) The different humanized mice are not adequately introduced to the reader, nor are the results discussed in the context of the different biology of these models in a lot of depth. Indeed, the acronymns are not spelt out even in the materials and methods and a search of the Jax catalogue for “Hu-BLT” yeilds no results (the other strains could be found, but are given their full name, which is what is required in the manuscript). The discussion touches on differences in GVH (but again this is not explained) in these mouse strains, but there are also difference in the types of cells that develop in these mice. Given the role of macrophages/monocytes as an important cell for dissemination of ectromelia virus, the fact that these cells tend not to develop well in Hu-PBMC mice also be significant. | We have added info on how these mice are generated in intro section and provided a reference for HU-BLT mice creation from UMASS (as JAX no longer makes that model). GVH is a limitation of this model when testing MCMs and we have added a reference. We feel that doing a serial sacrifice study, where early time points are taken, would be help in addressing the human cell(s) needed for infection. Have added in discussion on ECTV findings and how similar in vitro studies could be informative for VARV. |
|  |  |
| Reviewer #3: Line 89. It is mentioned that hu-BLT mice inoculated with low dose group succumbed to disease, but hu-CD34 mice also succumbed at low dose (Table S1 and Fig.1). | Thank you for noting this, we have updated (line 189) |
|  |  |
|  |  |
|  |  |
| I could not see the legends to Supplementary Figures. | Updated |
|  |  |
| Table S1 should be included in the manuscript since it has relevant information. | Moved to manuscript main text. |
|  |  |
