# Supplementary material for: Teaching a new mouse old tricks: Humanized mice as an infection model for Variola virus
Source: PLoS Pathog. 2021 Sep 21;17(9):e1009633. doi: 10.1371/journal.ppat.1009633 (PMC8454956; doi:10.1371/journal.ppat.1009633)
Supplement: Attachment — Submitted filename: Round 2 reviewer comments.docx [file ppat.1009633.s005.docx]

Thank you for the additional suggestions. We have responded in a point-by-point manner below and specifically indicated where in the manuscript (when looking at version with Track Changes) the changes were made.

We would also like to suggest a change in the title as humanized mice are an infection model for variola virus rather than a model for variola virus as stated. Also. rather than questioning whether you can teach a new mouse old tricks, you might change the title to the more affirmative "Teaching a new mouse old tricks: Humanized mice as an infection model for variola virus" as an example.

Yes, thank you for the suggestion we have made this change to the title.

In this revised manuscript, the authors have addressed most of the issues raised about the presentation of the data (noting some exceptions below), but not the substantive point made in my original review that the study remains limited by the very small group sizes and lack of replication, neither was this rebutted in any way. There remains no replication of the experiments and no argument was made as to why this is not necessary.

While we agree that replication of the study with larger group sizes would be beneficial, this is unfortunately not feasible. Studies in BSL4 laboratories are costly and intensive, and for work with Variola virus must have direct public health impact and be approved by WHO. With this pilot study, we have shown that the virus is able to spread systemically resulting in death of the animals. Due to the strict regulatory oversight of work with this virus, the model is not meant to be used for large-scale GLP studies for testing of medical countermeasures. Rather, it may be used as an early tool for testing of promising new smallpox therapeutics directly against Variola virus. This initial study established that the model may be used for this purpose; therefore it is unlikely that WHO will approve a repeat study with larger number of animals. However, based on the pilot study results, we have obtained approval from WHO for subsequent serial sacrifice studies to better characterize trafficking of the virus in the mouse strains. These studies will be useful in better characterizing the model and answering some of the excellent questions that the reviewers and editor have raised.

The new presentation of the virus titers further reinforce that variation is a substantial issue here that may make these models difficult to use. Interestingly the HuCD34+ mice seemed more promising in this regard with relatively consistent titers from most tissues rather than viral loads spanning many orders of magnitude as seen with the other types of mice.

Thank you for this suggestion. We have added an additional paragraph to discussion (starting on line 327). It is important to note that while there were variations in virus titers (more notably in the BLT mice), all infected BLT and HuCD34+ mice uniformly succumbed to infection.

1) The intranasal route of infection should be noted in the first sentence describing the experiment in the Results section and in at least the legend for the first figure.
   a. We have updated as requested in line 101 and Figure legend.
2) An outline of the experiment as a panel in the first figure showing a timeline with infection, starting numbers, when mice succumbed and sampling time would also make things clearest.
   a. We have updated Figure 1 with this suggestion and updated Figure legend (starting line 692).

2) Statistical comparisons made in the text remain difficult to assess from the data shown in figures because often the groups being compared are separated on different graphs. E.g. a statistical comparison of mortality is made between doses for each models in the text, but the groups are plotted on two sets of axes in the figure, with the two doses on separate graphs.

We have combined the mortality curve into one figure (within Figure One) to make the comparison easier on the reader. During the previous review, reviewer asked us to separate out in Table 2 by mouse strain and dose, which was done to make similar to Table 1.

3) The authors still use “n” to refer both to the total group size and the number of mice with a particular finding or are ambiguous in numerical descriptions. In one case this is “n=2/8”. In another sentence “involving up to 50% of liver in hu-CD34+ (n=3) and hu-BLT (n-=5)” – does this mean 50% of the livers in affected mice, or livers in 50% of the mice (but if the group sizes are 3 and 5, how does this make sense). The use of “n” should be exclusively for the group size. When reporting findings “1/5” as used in some places (or 1 of 5) should be used consistently. The reader should not be having to go to the tables to make sense of the text.

We have tried to clarify and make more consistent throughout the text including in lines 132-135. 191-192. 199.

4) Do the virus loads correlate in any way to clinical findings? This should be explored and discussed.

We have compared the virus loads and clinical signs to see if there is any correlation. Hu-CD34s and BLTs were euthanized prior to day 21 due to clinical signs. Since they were all euthanized it is difficult to make this correlation (vs if we had animals that became sick and recovered). We did look at the viral loads in those animals that were found dead vs. euthanized and there were no obvious trends (not shown in manuscript). However, for PBMC mice, the one animal that was euthanized on 19 dpi due to clinical signs did on average have higher titers than other animals in that group. We added this info to the manuscript (Line 207-208).

5) Variation in the data should be discussed as this has an impact on the usability of these models to test drugs etc.

As noted above we have added this discussion on the viral titer variation seen to the discussion (starting on line 327). However, there was NO variation in that all infected animals (BLT and Hu-CD34) succumbed to infection. In the High dose challenge groups, the timeline until death was similar (for both CD34 and BLT) within the mouse strain type. We also did statistics comparing CD34 and BLT viral loads and nothing was statistically significant The main clinical score for this model is weight loss which is an objective measure. We added that point at line 110.
